# Supplementary material for: Association between oral microbiome and seven types of cancers in East Asian population: a two-sample Mendelian randomization analysis
Source: Front Mol Biosci. 2023 Nov 21;10:1327893. doi: 10.3389/fmolb.2023.1327893 (PMC10702768; doi:10.3389/fmolb.2023.1327893)
Supplement: Supplementary file 1 [file DataSheet1.ZIP › Supplementary Materials/MR plots of saliva/saliva═╝/colorectal cancer/pheno.913_to_colorectal cancer_forest.pdf]

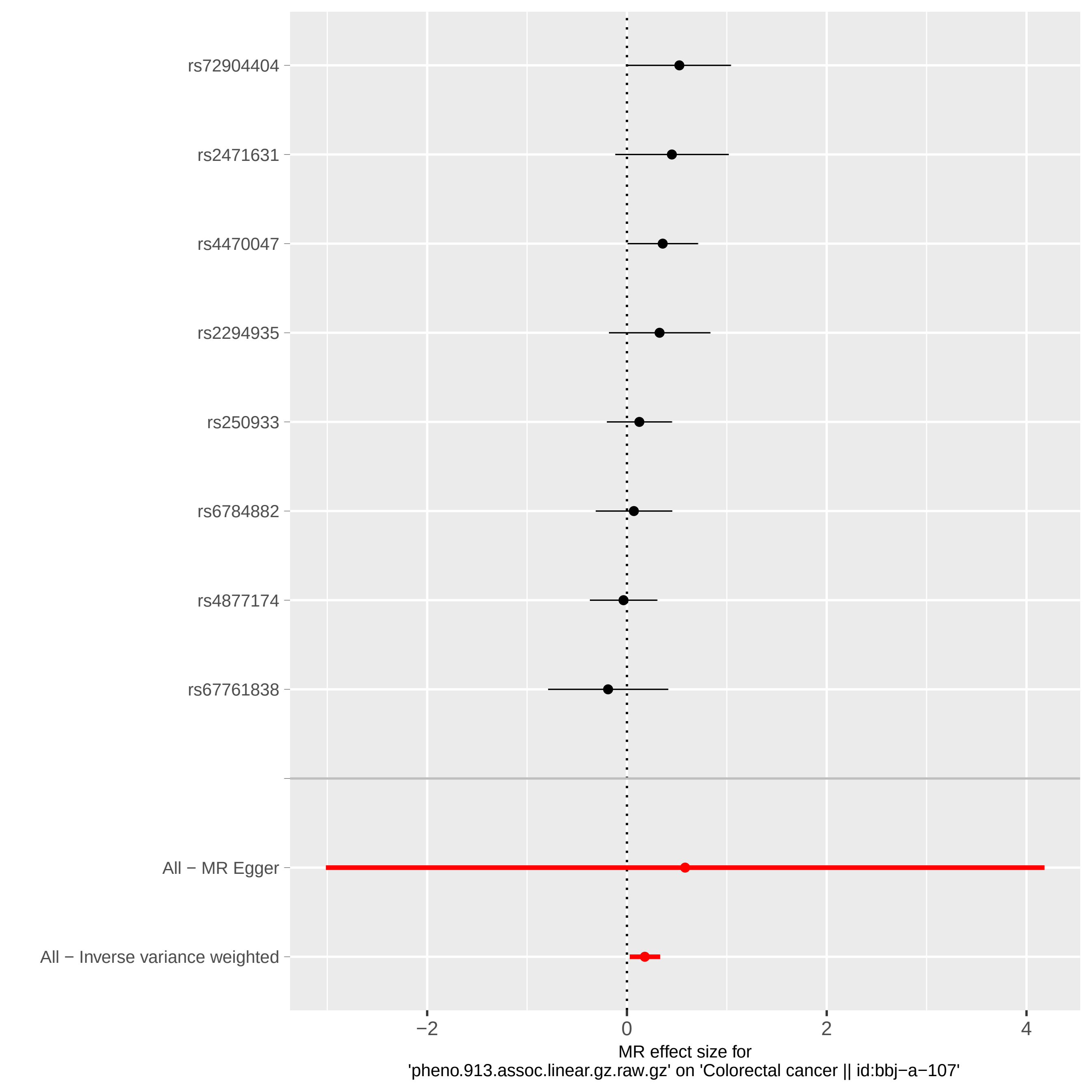

rs72904404
rs2471631
rs4470047
rs2294935
rs250933
rs6784882
rs4877174
rs67761838
All − MR Egger
All − Inverse variance weighted
−2
0
2
4
MR effect size for
'pheno.913.assoc.linear.gz.raw.gz' on 'Colorectal cancer || id:bbj−a−107'
